# Supplementary material for: Tobacco smoking initiation among students in Samoa and health concerns
Source: PLoS One. 2021 Oct 29;16(10):e0258669. doi: 10.1371/journal.pone.0258669 (PMC8555831; doi:10.1371/journal.pone.0258669)
Supplement: Attachment — Submitted filename: Response to Reviewers.docx [file pone.0258669.s001.docx]

**Response to Reviewers’ comments**

We are very thankful to the reviewers for their detailed and insightful comments and suggestions. It has helped us to improve the manuscript significantly.

**Comments to the Author**

1. Is the manuscript technically sound, and do the data support the conclusions?

The manuscript must describe a technically sound piece of scientific research with data that supports the conclusions. Experiments must have been conducted rigorously, with appropriate controls, replication, and sample sizes. The conclusions must be drawn appropriately based on the data presented.

Reviewer #1: Partly

This study is based on survey data, which was collected through scientific methodology developed by World Health Organization (WHO). This is a normal practice in this kind of studies. We have done some revisions to the analysis section to provide a more technical tone to the script. We have used data collected by WHO.

Reviewer #2: Yes

2. Has the statistical analysis been performed appropriately and rigorously?

Reviewer #1: N/A

The statistical analysis has been conducted using the multinominal logistic regression which is robust across different scenarios. In addition to this, we have also performed the Hosmer-Lemeshow Goodness-of-Fit test to show the excellent fit of the regression model. The test overall shows that the model fits the data well from which the discussions are outlayed.

Reviewer #2: Yes

3. Have the authors made all data underlying the findings in their manuscript fully available?

The [PLOS Data policy](http://www.plosone.org/static/policies.action#sharing) requires authors to make all data underlying the findings described in their manuscript fully available without restriction, with rare exception (please refer to the Data Availability Statement in the manuscript PDF file). The data should be provided as part of the manuscript or its supporting information, or deposited to a public repository. For example, in addition to summary statistics, the data points behind means, medians and variance measures should be available. If there are restrictions on publicly sharing data—e.g. participant privacy or use of data from a third party—those must be specified.

Reviewer #1: Yes

Reviewer #2: Yes

4. Is the manuscript presented in an intelligible fashion and written in standard English?

PLOS ONE does not copyedit accepted manuscripts, so the language in submitted articles must be clear, correct, and unambiguous. Any typographical or grammatical errors should be corrected at revision, so please note any specific errors here.

Reviewer #1: Yes

Reviewer #2: Yes

5. Review Comments to the Author

Please use the space provided to explain your answers to the questions above. You may also include additional comments for the author, including concerns about dual publication, research ethics, or publication ethics. (Please upload your review as an attachment if it exceeds 20,000 characters)

Reviewer #1: This study is interesting and have a great impact on public health threat, especially for young students in Samoa and the Pacific Island Countries. The data also insight on the world economy and policy making suggestions.
Right now, the whole world is affected with COVID-19 infection and study reported that young smokers were more got infected with COVID-19 compared with non-smokers.
1. How does COVID-19 infection relate to the mortality rate with young smoker VS Non-smokers

We have cited and included some papers analyzing on the above question in this paper.

Prats-Uribe, Xie (2021) find no difference in mortality rate between young smokers and non-smokers, however young smokers were twice likely to get infected with serious COVID-19 illness compared to non-smokers, an important finding from the United States.

2. How does the recovery from COVID-19 infection affected the young smoker?

To our knowledge, there is no studies on long-term illness and impact of smoking on recoveries of illness from COVID-19, nevertheless, the higher risk of young smokers of been medically vulnerable, the medical policy intervention to address smoking among young smokers is likely to decrease their medical vulnerability to severe illnesses. This part as been included in the paper.

Reviewer #2: In this manuscript “Tobacco Smoking Initiation among students in Samoa and Health Concerns“ Singh et al. demonstrated a living problem of socio-economic, education and culture in context of tobacco smoking in the young generation of Samoa.
The study has a significant impact apropos of socio-economic background, which is not only a problem of a particular area but it is the issue of many countries in all over the world.
Here, authors demonstrated how home and friend circle environment, parental smoking, pocket money and mother’s education have an impact on youths in smoking in Samoa. They used empirical formula for the analysis: multinomial logit regression.
However, I have few minor question:
1) Introduction is too long, authors can decrease the volume.

As recommended, we have reduced the size of the introduction section by removing few sentences and condensing main ideas.

2) In table -1, sex, age should be bold

As recommended, we have made the above changes in the paper.

3) Authors used empirical model in the method section, they should mention the use of software/link.

Authors have used Stata for conducting statistical analysis. This information has been included in the empirical model section.

4) In conclusion, in a few lines, authors may emphasize and write their main findings and followed by how to overcome that problem.

6. PLOS authors have the option to publish the peer review history of their article ([what does this mean?](https://journals.plos.org/plosone/s/editorial-and-peer-review-process#loc-peer-review-history)). If published, this will include your full peer review and any attached files.


If you choose “no”, your identity will remain anonymous but your review may still be made public.


**Do you want your identity to be public for this peer review?** For information about this choice, including consent withdrawal, please see our [Privacy Policy](https://www.plos.org/privacy-policy).

Reviewer #1: No

Reviewer #2: No
